# The surface ectoderm exhibits spatially heterogenous tension that correlates with YAP localisation during spinal neural tube closure in mouse embryos

**DOI:** 10.1016/j.cdev.2023.203840

**Published:** 2023-06

**Authors:** Abigail R. Marshall, Gabriel L. Galea, Andrew J. Copp, Nicholas D.E. Greene

**Affiliations:** Developmental Biology and Cancer Department, UCL Great Ormond Street Institute of Child Health, University College London, UK

**Keywords:** Neural tube closure, Surface ectoderm, Biomechanics, Laser ablation

## Abstract

The single cell layer of surface ectoderm (SE) which overlies the closing neural tube (NT) plays a crucial biomechanical role during mammalian NT closure (NTC), challenging previous assumptions that it is only passive to the force-generating neuroepithelium (NE). Failure of NTC leads to congenital malformations known as NT defects (NTDs), including spina bifida (SB) and anencephaly in the spine and brain respectively. In several mouse NTD models, SB is caused by misexpression of SE-specific genes and is associated with disrupted SE mechanics, including loss of rostrocaudal cell elongation believed to be important for successful closure. In this study, we asked how SE mechanics affect NT morphology, and whether the characteristic rostrocaudal cell elongation at the progressing closure site is a response to tension anisotropy in the SE. We show that blocking SE-specific E-cadherin in *ex utero* mouse embryo culture influences NT morphology, as well as the F-actin cable. Cell border ablation shows that cell shape is not due to tension anisotropy, but that there are regional differences in SE tension. We also find that YAP nuclear translocation reflects regional tension heterogeneity, and that its expression is sensitive to pharmacological reduction of tension. In conclusion, our results confirm that the SE is a biomechanically important tissue for spinal NT morphogenesis and suggest a possible role of spatial regulation of cellular tension which could regulate downstream gene expression *via* mechanically-sensitive YAP activity.

## Highlights

• Disruption of surface ectoderm E-cadherin affects spinal neural tube morphology.
• Elongated surface ectoderm cell shape is not due to tension anisotropy.
• Surface ectoderm tension is regionally heterogenous as revealed by laser ablation.
• YAP nuclear translocation reflects differences in surface ectoderm tension.

## 1 Introduction

The vertebrate neural tube (NT) is the embryonic precursor of the brain and spinal cord, and consists of neuroepithelium (NE) with overlying surface ectoderm (SE). Both are crucial to the formation of the NT, by a process termed neurulation or neural tube closure (NTC). During NTC a flat sheet of pseudostratified NE, the neural plate, undergoes folding, elevation of bilateral neural folds and midline adhesion and tissue fusion to form a complete NT with a single cell layer of overlying SE. In mammals, NTC occurs as the embryo continues to elongate, with fusion of the neural fold tips occurring progressively from a number of initiation sites, a process referred to as ‘zippering’. The most recently closed point of the NT is referred to as the ‘zippering point’ (ZP). Neurulation completes with the closure of neuropores, the last of which to close at approximately embryonic day (E)10.5 in the mouse is the posterior neuropore (PNP) at the caudal extremity of the embryo. Failure of NTC leads to a group of congenital disorders known as neural tube defects (NTDs), the most common of which are anencephaly (affecting the future brain) and spina bifida (SB, or myelomeningocele) which results from failure of PNP closure (Greene and Copp, 2014).

The NE is commonly thought of as the mechanical ‘driver’ of the morphological movements of NTC, because early shaping, folding and elevation are controlled by cellular mechanisms within the NE (Nikolopoulou et al., 2017). However, it has become increasingly clear that the SE is also required for successful closure. For example, the SE is the source of BMP signalling whose inhibition is required for dorsolateral bending of the NE in the lower spine (Ybot-Gonzalez et al., 2007); just before fusion, the apposing neural folds produce filopodia and lamellipodia which create the first contact, and it is from the SE that these protrusions originate (Rolo et al., 2016; Ray and Niswander, 2016; Mole et al., 2020); an F-actin rich cable encircling the open neuropore during hindbrain closure colocalises with SE borders and is an important pro-closure mechanism (Galea et al., 2021); finally, NTC fails in embryos carrying mutations in a number of SE-expressed genes, including the transcription factor-encoding genes *Grainyhead-like -2* and *-3* (Yu et al., 2006; Rifat et al., 2010; Brouns et al., 2011).

After NTC, the SE subsequently differentiates into epidermis, a process which is now understood to involve mechanical cues (Simpson et al., 2011; Kenny et al., 2018). This is partly controlled by the transcription factor Yes-associated protein (YAP), which maintains epidermal progenitors in their proliferative state and plays an important role in the timing of differentiation (Zhang et al., 2011; Totaro et al., 2017; Sedov et al., 2022). YAP is commonly used as a mechanical readout due to its changes in activity in response to substrate stiffness. YAP is translocated to the nucleus in conditions of high stiffness, which in turn increases actin cytoskeleton tension (Dupont et al., 2011); Rho-ROCK signalling, which increases contractility by regulating the cellular actomyosin network, also promotes YAP activity (Kono et al., 2014). Whether or how YAP may be involved in neurulation, which is highly dependent on mechanics, remains unknown.

NTC involves a precise balance of biomechanical forces both at the scale of the whole tissue and single cells. In the NE, inhibition of F-actin turnover leads to an accumulation of apical actomyosin, preventing spinal NTC in wild type mouse embryos, possibly due to an increase in neuroepithelial tension (Escuin et al., 2015). In addition, constriction of the NE in the open PNP brings the neural folds closer to the midline and promotes closure (Galea et al., 2021; Galea et al., 2017). At the cellular level, both NE and SE cells are required to disassemble cell-cell and cell-matrix contacts to create new contacts at the fusion point. In mouse embryos, loss of integrin β1 at the ZP prevents formation of a cell semi-rosette, which is important to promote fusion point progression (Mole et al., 2020). Other mouse models which fail to close their NT show abnormal cell shape on a wider scale, for example *Grhl2*^*−/−*^ embryos lose the characteristic rostrocaudal elongation of cells extending along the midline of the recently closed SE (Nikolopoulou et al., 2019).

It is predicted that cellular and tissue level biomechanics in the NE and SE must be coordinated for closure to succeed. Transmission of forces between the two tissues may be achieved by the formation of paired F-actin rich cables, which run along the SE-NE junction in the neural folds of the open PNP, connecting the constricting NE to the ZP (Galea et al., 2017). The F-actin cables are predicted to be important in PNP closure, as has been shown in closure of the hindbrain neuropore *via* a purse-string like mechanism (Maniou et al., 2021). However, the PNP cable does not encircle the PNP until the very end of closure and so cannot act as a ‘purse-string’ (Galea et al., 2017). Therefore, the mechanism by which it influences spinal neurulation is not known. Disruption of the PNP cable correlates with failed spinal closure in some models, but its presence is not sufficient for normal neurulation, showing the need for multiple contributory factors for propagation of closure (Mole et al., 2020; Galea et al., 2017; Nikolopoulou et al., 2019; Maniou et al., 2021; Butler et al., 2019).

We have previously begun to dissect the role of the SE in neurulation by studying how loss- and gain-of-function mutations of *Grhl2* lead to highly penetrant SB. We found that mutated *Grhl2* alters expression of genes encoding E-cadherin (*Cdh1*) and multiple claudins (*Cldn-3, -4, -6, -7 and -8*), simultaneously altering SE cell shape, actomyosin abundance and tissue tension at the ZP (Nikolopoulou et al., 2019). Pharmacological inhibition of claudins in mouse embryo culture also delays spinal NTC (Baumholtz et al., 2017). However, interpretation of these studies in the context of the specific role of the SE is complex, due to potential non-SE effects (pharmacological claudin inhibition also affects the NE) and multiple downstream transcriptomic effects caused by mutation of a transcription factor (*Grhl2*). There is therefore a need for more specific perturbations of SE function.

In the current study, we asked how short term blockade of E-cadherin function in whole embryo culture affects NT morphology. This confirmed that the SE is a biomechanically important tissue for PNP closure. We then further characterised the mechanical properties of the SE using single border laser ablation, cell shape and YAP expression analysis. Our findings suggest that tension is tightly controlled across the recently closed NT, and that this may in turn regulate mechanically-sensitive YAP activity.

## 2 Methods

### 2.1 Animal procedures

All mouse work was performed under regulations of the UK Animals (Scientific Procedures) Act 1986 and the Medical Research Council's Responsibility in the Use of Animals for Medical Research (1993). Mice were mated overnight and females checked for a copulation plug the next morning, which was designated embryonic day (E)0.5. Three strains of wild type mice were used: CD1 mice for whole embryo culture experiments, C57/BL6 mice for cell shape analysis, surface ectoderm laser ablations and YAP experiments, and BALB/c mice for additional YAP experiments. *Grhl2*^*Gt (AC0205)Wtsi*^ mice carry a gene-trap construct in *Grhl2* and were maintained on a BALB/c background (Brouns et al., 2011). Homozygous *Grhl2*^*Gt/Gt*^ is functionally null for *Grhl2*, as evidenced by lack of mRNA expression. Therefore, heterozygotes and homozygotes are referred to as *Grhl2*^*−/+*^ and *Grhl2*^*−/−*^ respectively. Yolk sacs were collected for genotyping as previously described (Brouns et al., 2011).

### 2.2 Embryo dissection

Embryos were collected at developmental stages from E8.5–9.5. Individual deciduas were dissected from the uterus in Dulbecco's Modified Eagle's Medium (DMEM) + 10 % fetal bovine serum (FBS), pre-warmed to 37 °C. Embryos for immunostaining were removed from their membranes and fixed in 4 % paraformaldehyde (PFA) overnight.

### 2.3 Embryo culture and microinjection

For embryo culture, the decidua, trophoblast and Reichert's membrane were carefully removed, leaving the intact yolk sac attached to the ectoplacental cone. Embryos were ranked according to size of the yolk sac and distributed evenly between experimental and control groups. For microinjection experiments, sterile PBS, with or without E-cadherin blocking antibody (Invitrogen, 16324982), was mixed with a small volume of CellMask Deep Red plasma membrane (C10046 Invitrogen) for visualisation. This functional antibody clone (DECMA-1) has been shown to inhibit E-cadherin-dependent pathways *in vivo* and *in vitro*: in a mouse model of neuropathic pain, intrathecal injection of DECMA-1 inhibits an E-cadherin dependent increase in p120ctn, reducing the anti-nociceptive effects of the molecule GDNF (Wang et al., 2014); in MDCKII epithelial cells, culture with DECMA-1 prevents cells from adhering from each other and disrupts Rac1 localisation (Nakagawa et al., 2001). A mouth-controlled micropipette was used to inject the solution directly into the amnion until it was filled, as judged by dye overflowing back into the medium. Embryos were cultured in rat serum (0.5 mL per embryo) in 25 mL Nunc Universal tubes (Thermo Scientific, 364238) gassed with 5 % CO_2_ and either 5 % O_2_/90% N_2_ (E8.5–9.5) or 20 % O_2_/75% N_2_ (>E9.5), and incubated at 37 °C on a rolling apparatus. 10 μM Y27632 ROCK-inhibitor (Cambridge Biosciences, SM02-1) or PBS were added directly to the rat serum. Embryos which failed to turn during the 24 h culture, and/or did not have an intact yolk sac circulation, were excluded from further analysis. This comprised: 0/20 non-injected, 4/17 vehicle-injected and 5/23 E-cadherin antibody-injected embryos.

### 2.4 Laser ablation

Laser ablation experiments were carried out as previously described (Maniou et al., 2021; Marshall et al., 2022). Each decidua was transferred to an individual 1.5 mL Eppendorf in DMEM + FBS, gassed with 5 % CO_2_, 20 % O_2_ and 75 % N_2_ and kept on a hot block at 37 °C. Embryos were processed one at a time to minimise time out of temperature control. Each embryo was dissected from all membranes and stained for 5 min at 37 °C with 1:500 CellMask in DMEM + FBS. The caudal half of the embryo was then separated from the rostral half, as the beating heart would prevent accurate ablations of single cell borders. The caudal half was fixed into place in media in agarose-coated petri dishes using microsurgical needles (11–0 Mersilene; TG140-6; Ethicon and 10–0 Prolene; BV75-3; Ethicon). For ablation and imaging, a single z-slice of the target region was imaged for 2 frames, ablated, and imaged for a further 18 frames (each frame ~0.9 s). For imaging, the laser power was varied depending on the CellMask signal, while the laser power for the ablations was always 100 % with a pixel dwell time of 0.34 μs. 71 embryos were ablated 2–3 times (including mediolateral and rostrocaudal ablations), although not all ablations were included in the analysis (see Image analysis below). Borders which were flat within the z plane of imaging were prioritised for ablations, as this made landmark-based analysis of recoil easier. Ablations oriented up to 45° from the rostrocaudal axis were ablated with a mediolaterally oriented cut, while ablations up to 45° from the mediolateral axis were ablated with a rostrocaudally oriented cut.

### 2.5 Immunofluorescence

For whole mount immunofluorescence, embryos fixed in 4 % PFA were washed 3× with PBS before blocking and permeabilisation in blocking solution (PBS + 0.1 % Tween + 4 % bovine serum albumin (BSA)) overnight at 4 °C. The next day, embryos were incubated overnight in primary antibody diluted in blocking solution. Primary antibodies and respective concentrations were: E-cadherin (BD Biosciences 610181, 1:200); ZO1 (Invitrogen 402200, 1:100); YAP (Cell Signalling 14704, 1:100). The next morning, embryos were washed 3 × 1 h in blocking solution, with shaking at room temperature. Secondary antibodies (Alexa Fluor®, Thermo Fisher Scientific) were added at a concentration of 1:500 in blocking solution, along with 1:5000 DAPI, and incubated for two hours with shaking at room temperature and protected from light. When used, 1:50 Phalloidin-647 (Thermo-Fisher Scientific, A22287) was included in the secondary antibody solution. Embryos were washed 3 × 1 h in PBS and stored in PBS + 1 % sodium azide.

### 2.6 Image analysis

Images were obtained using a Zeiss Examiner LSM880 confocal microscope, using either a 10×/NA0.5 W-Plan Apochromat or 20×/NA1.0 Plan Apochromat dipping objective.

Confocal images of whole mount-stained embryos were segmented using an in-house generated macro as previously described (Galea et al., 2021; Galea et al., 2018), in order to remove fluorescence signal from the underlying neuroepithelium (‘surface-subtracted’) and allow analysis of signal from the surface ectoderm. This macro is available courtesy of Dr. Dale Moulding on GitHub (https://github.com/DaleMoulding/Fiji-Macros). All image analysis was carried out on z-projections of surface-subtracted images. Cell shape was analysed using an inbuilt FIJI plugin, TissueAnalyzer (Aigouy et al., 2016).

To quantify nuclear intensity of YAP and DAPI, nuclei were segmented and binarised using a morphological filter from the inbuilt FIJI plugin MorphoLibJ (Legland et al., 2016). Particles with area 2–100 μm^2^ and circularity 0.6–1 were selected from the binarized image. Finally, the 3D objects counter plugin was used, with intensity measurements redirected to YAP and DAPI channels, thus giving an average fluorescence readout for each individual nucleus (Bolte and Cordelières, 2006).

F-actin cable intensity was analysed in FIJI by drawing a mediolaterally-oriented line of constant length through the ZP. The fluorescence profile of this line was then measured, and the peak intensity (corresponding to the brightest point of the cable at the ZP) was compared to the average intensity of the fluorescence intensity profile.

Recoil analysis of single cell laser ablations was carried out by measuring the distance between two reference points before and after ablation. Ablations which hit two or more cell borders, and images which showed significant blebbing (indicating embryo damage) were excluded from analysis.

Particle image velocimetry (PIV) analysis involved applying a bandpass filter to the image, followed by application of the inbuilt FIJI PIV plugin (Tseng et al., 2012).

### 2.7 Statistical analysis

All statistical tests were carried out, and all graphs generated, using Origin Lab versions 2017–2021 (OriginLab, Northampton, MA). Parametric data was plotted as mean +/− S.D. Non-parametric data was displayed as box plots.

## 3 Results

### 3.1 Surface ectoderm biomechanics influence PNP morphology

**Fig. 1:**
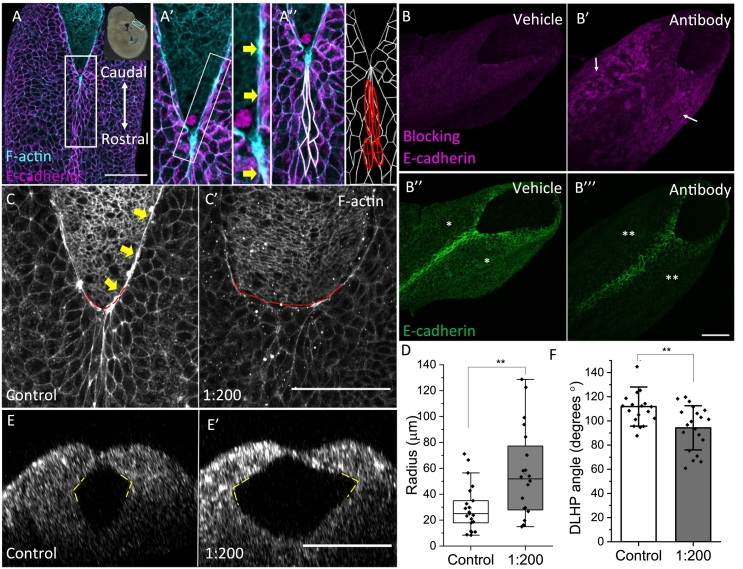
Surface ectoderm mechanics directly affect posterior neuropore morphology. A) Whole mount immunofluorescence for E-cadherin and F-actin in the closing spinal NT of an E9.5 mouse embryo (inset at top right in A, PNP outlined in cyan). A′, A″) Enlargements of the boxed region in A. An F-actin rich cable runs along the open neural folds of the PNP (yellow arrows in A′, right image). SE cells in the recently closed NT are rostrocaudally elongated (A″; outlined in white, fitted ellipses in red). B) Whole mount immunofluorescence using a secondary antibody to detect bound E-cadherin blocking antibody (B, B′; magenta) and use of a non-blocking primary antibody to detect endogenous E-cadherin (B″, B‴; green). Embryos were fixed after 1 h in culture following injection with vehicle (B, B″) or E-cadherin blocking antibody (B′, B‴). Note the presence of bound E-cadherin blocking antibody, particularly on SE cells lateral to the midline (arrows in B′). This coincides with depletion of endogenous E-cadherin (compare * and ** in B″ and B‴ respectively). C) F-actin immunostaining reveals that the F-actin cable in 1:200 treated embryos (C′) is disrupted compared to controls (yellow arrows in C). The V-shaped ZP (dashed red line) is also disrupted in blocking antibody-treated embryos compared to controls. D) Quantification of the ZP shape change in C) by measuring the radius of a fitted circle at the ZP, which shows a significantly larger curvature of the treated ZP compared to controls (** Mann-Whitney test, p < 0.01, n = 39 embryos). E, F) The DLHP angle (average of both angles, yellow dashed lines) of embryos treated for 24 h with E-cadherin blocking antibody (1:200) was significantly smaller compared to controls (mean +/− SD, ** *t*-test, p < 0.004, n = 39 embryos). E and E′ obtained by reflection imaging and subsequent reslicing in the YZ plane. All scale bars = 100 μm.

**Fig. 2:**
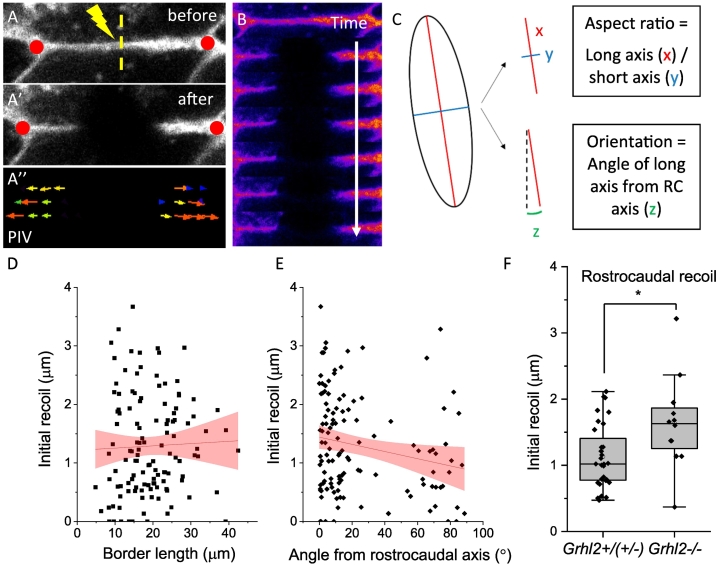
Single border laser ablation reveals that cell shape is not reflective of tension anisotropy. A, A′) Example of cell border recoil before (A) and after (A′) laser ablation. Yellow dashed line = site of ablation. CellMask is used to visualise borders. Red dots show example reference regions which are used to measure recoil, by measuring the distance between points before and after ablation. A″) Example particle image velocimetry (PIV) of the ablated region from A) showing lateral border recoil away from the ablation site. The direction of arrows represents the direction of tissue movement caused by recoil. B) Kymograph of ablated cell border from A) over a period of ~7 s, showing a stable ablation which does not recover over time. C) Schematic to show how fitted ellipses for cells are used to analyse cell shape. Aspect ratio is calculated by dividing the length of the long axis (x, red) by the length of the short axis (y, blue). The angle of the long axis (z, green) is measured compared to the rostrocaudal axis (black dotted line, angle = 0°). D, E) Border length (D) does not correlate with SE recoil, while there is a negative correlation between angle from rostrocaudal axis and recoil (E). Red shading = 95 % confidence interval around best-fit linear regression lines. Length: r^2^ = 0.001, F-test, p = 0.7. Angle: r^2^ = 0.04, F-test, p = 0.02. n = 122 ablations. F) Mutant *Grhl2−/−* SE cell borders show significantly higher rostrocaudal recoil than wild type borders (* Mann-Whitney test, p < 0.02, n = 45 embryos).

The neural folds of the open PNP have a characteristic F-actin cable localised in the SE cells at the boundary with the NE, as shown by E-cadherin and F-actin colocalisation (Figs. 1A, A′, S2A (Maniou et al., 2021)). Immediately rostral to the open PNP, midline cells in the most recently closed SE are elongated and oriented along the rostrocaudal axis (Figs. 1A″, 2C, S1A – E), a characteristic property which is lost in association with spinal NTDs in some models, including *Grhl2* loss of function (Nikolopoulou et al., 2019). Here, we investigated the relationship between biomechanical properties of the SE (*i.e.* the F-actin cable, cell shape) and morphology of the PNP to determine how these may relate to progression of spinal NTC.

*Cdh1* (which encodes E-cadherin) is among a series of cell junction protein-encoding genes that are down-regulated in mouse embryos with loss of function of *Grhl2* (Nikolopoulou et al., 2019). To specifically assess the requirement for E-cadherin in PNP closure, we inhibited its function in mouse embryo culture using a function-blocking antibody. Initially, we tested binding of the antibody to the SE by injecting E9.5 embryos with either PBS alone, or PBS + 1:50 blocking antibody, followed by 1 h culture and immediate fixation. Binding of the blocking antibody was detected using a secondary antibody, which shows extensive signal in the SE lateral to the midline of antibody-injected embryos (Figs. 1B′, S2D) but not in vehicle-injected controls (Figs. 1B, S2B). Staining of the same embryos with a non-blocking primary E-cadherin antibody reveals diminished binding to E-cadherin in SE cells lateral to the midline of blocking antibody-treated embryos (Figs. 1B‴, S2D), compared with vehicle-injected controls (Fig. 1B″), and this correlates closely with the main site of blocking antibody visualised (Fig. 1B, B′). Hence, binding of the blocking antibody appears to deplete SE cells of E-cadherin. The rostrocaudal midline of both treated and control embryos show persistent E-cadherin signal (Figs. 1B″, B‴, S2B, C), and this coincides with reduced presence of blocking antibody (Figs. 1B′, S2B–C), perhaps suggesting higher turnover of E-cadherin, together with bound blocking antibody, in midline SE cells.

Having demonstrated specific binding of the blocking antibody, and depletion of E-cadherin in the SE, additional cultures were carried out for 24 h from E8.5. A lower blocking antibody concentration was used for these experiments (1:200) to avoid embryo toxicity, and indeed, we did not find any adverse effect on embryo developmental progression, as measured by somite gain (Fig. S3A). Embryos treated with the blocking antibody show a markedly disrupted F-actin cable after culture (Figs. 1C, C′, S3B), a similar phenotype to *Grhl2*^*−/−*^ mutants in which spinal NTC fails (Nikolopoulou et al., 2019). Quantification of the peak actin fluorescence intensity at the ZP compared to the average intensity confirms a significant reduction in actin accumulation at the cable (Fig. S3B–C). In addition, instead of the V-shaped ZP observed in dorsal views of vehicle-treated controls, these embryos show a more rounded ZP morphology, another phenotype observed in *Grhl2*^*−/−*^ mutants (Fig. 1C, C′). This apparent shape change was confirmed by quantifying the radius of a circle drawn at the ZP. This revealed significantly higher curvature in E-cadherin blocked embryos compared to controls (Fig. 1D), corresponding with more widely spaced neural folds. PNP length, which is indicative of rostrocaudal progression of the ZP, is comparable between treatment groups (Fig. S3D).

In addition to the shape of the ZP at the dorsal aspect of the neural folds, we asked whether PNP morphology was also altered in the NE not directly in contact with the SE. To test this, we measured the angle of the dorsolateral hinge points (DLHPs) which are bending sites within the NE. Loss of DLHPs has previously been shown to correlate with delayed PNP closure in the *Zic2*^*Ku/Ku*^ model (Ybot-Gonzalez et al., 2007), although a direct link between SE mechanics and DLHP morphology has not been demonstrated (Ybot-Gonzalez et al., 2007). Embryos cultured with the 1:200 dilution of E-cadherin blocking antibody show significantly more acute DLHP angles compared to controls (Fig. 1E–F), and these embryos also have a larger ZP curvature (Fig. S3E). Control embryos do not show a correlation between DLHP angle and curvature, showing that curvature and DLHP disruption is a consequence of E-cadherin blockade and not experimental manipulation (data not shown). Collectively, these findings suggest that the SE influences morphology of the PNP, either as a consequence of or in addition to disrupted development of the F-actin cable.

### 3.2 Laser ablation reveals regional heterogeneity in surface ectoderm tension

Having shown that disrupting the SE influences PNP morphology, we further probed mechanical properties of the wild type SE. Midline SE cells rostral to the ZP are rostrocaudally elongated (Figs. 1, S1), a characteristic of the PNP which is disrupted in some models of spinal NTDs (Mole et al., 2020; Nikolopoulou et al., 2019). However, the mechanism by which these cells become elongated is not known. One hypothesis is that cell tension in the wild type ZP region is anisotropic, *i.e.* higher in a rostrocaudal direction compared to mediolaterally. To test this, we carried out single cell border laser ablation, a technique which creates stable cuts in cell membranes and allows initial cell border recoil to be measured as a readout of tension (Fig. 2A, B, (Maniou et al., 2021; Marshall et al., 2022)). First, we analysed the relationship between cell orientation and recoil in wild type embryos. We predicted that longer cells (with greater aspect ratio, Fig. 2C) which are oriented parallel to the rostrocaudal midline would have higher recoil than mediolaterally oriented cells. However, surprisingly there is no correlation between recoil and cell length (Fig. 2D), although a weak negative correlation between orientation and recoil is observed (Fig. 2E).

We next examined the effect of *Grhl2* loss of function on cell border recoil by analysing non-elongated cells in *Grhl2*^*−/−*^ mutant embryos in the midline rostral to the ZP at E9.5, expecting that these non-elongated cells would show lower rostrocaudal recoil compared to elongated SE cells in wild type embryos (Fig. S4A, B). Surprisingly, the opposite is found, with non-elongated *Grhl2*^*−/−*^ SE cells showing significantly higher rostrocaudal recoil compared with wild type cells (Fig. 2F). These results led us to reject our initial hypothesis that anisotropic tension is responsible for rostrocaudal SE elongation. Instead, the data suggest that SE cell elongation is not due to preferential rostro-caudal stretching from the caudally-advancing ZP, but reflects intrinsic cell properties, such as cell rearrangement.

Having examined the ZP region at E9.5, we asked how tension may vary in the SE temporally and spatially. We began by analysing whether tension at the ZP changes with developmental stage during E9.0 - late E9.5, and found that there is no difference in rostrocaudal recoil in embryos with increasing numbers of somites within this period (Fig. S5). This result was confirmed by comparing recoil by somite stage in *Grhl2*^*+/+*^ and *Grhl2*^*+/−*^ embryos from the previous experiment (data not shown).

**Fig. 3:**
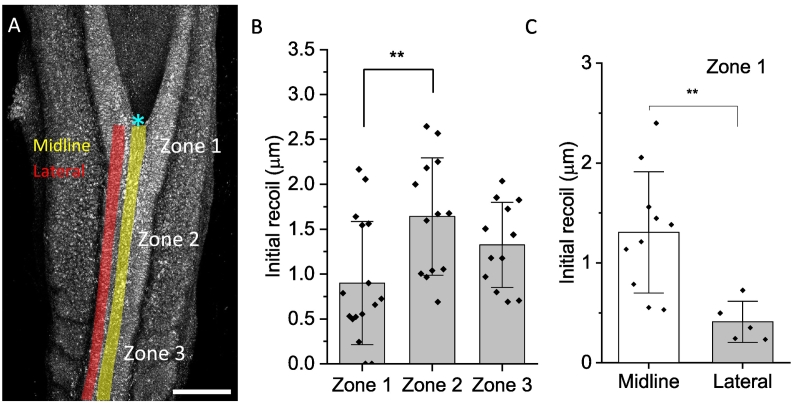
Surface ectoderm tension is regionally heterogenous. A) E9.5 mouse caudal region showing the different sites of SE ablation. Zone 1 = zippering point, Zone 2 = adjacent to the presomitic mesoderm, Zone 3 = adjacent to the somite region. Shaded yellow region = midline. Shaded red region = lateral. Cyan asterisk = ZP. Scale bar = 150 μm. B) Rostrocaudal recoil in Zone 1 is significantly lower than in Zone 2 (mean +/− SD, ANOVA, ** post-hoc Tukey's test, p < 0.008, n = 46 embryos). C) Midline cells in Zone 1 recoil significantly more than lateral cells (mean +/− SD, ** *t*-test, p < 0.008, n = 22 embryos).

We also compared different regions of the SE by analysing recoil of cells according to their rostrocaudal position with respect to the ZP. SE cells originate on either side of the PNP overlying the open neural folds, before becoming incorporated into the closed region as the ZP progresses caudally. Therefore, rostral SE cells are those which have been incorporated into the closed region at an earlier stage than caudal SE cells. To compare recoil between regions, we divided the SE into three separate zones (Fig. 3A): directly rostral to the ZP (Zone 1), further rostrally where somites had not yet formed (Zone 2), and yet further rostrally in the region adjacent to newly formed somites (Zone 3). Of note, the midline SE cell elongation seen in Zone 1 is not observed in Zones 2 and 3. Surprisingly, rostrocaudal recoil in Zone 1 is significantly lower than in Zone 2, and tends towards a significant difference compared to Zone 3 (Fig. 3B). Having observed lower midline recoil in Zone 1, we also tested whether medial and lateral regions in this region showed differences in recoil (shaded regions in Fig. 3A). Indeed, midline SE cells over the dorsal closed NT show significantly higher recoil compared to lateral cells (Fig. 3C). Overall, our results show that tension is regionally heterogenous; it is not yet known whether tension is regulated to keep ZP tension lower than baseline, or whether tension increases in SE cells that have passed the ZP as their cell-cell and cell-ECM adhesions mature.

### 3.3 YAP nuclear translocation is sensitive to surface ectoderm tension

**Fig. 4:**
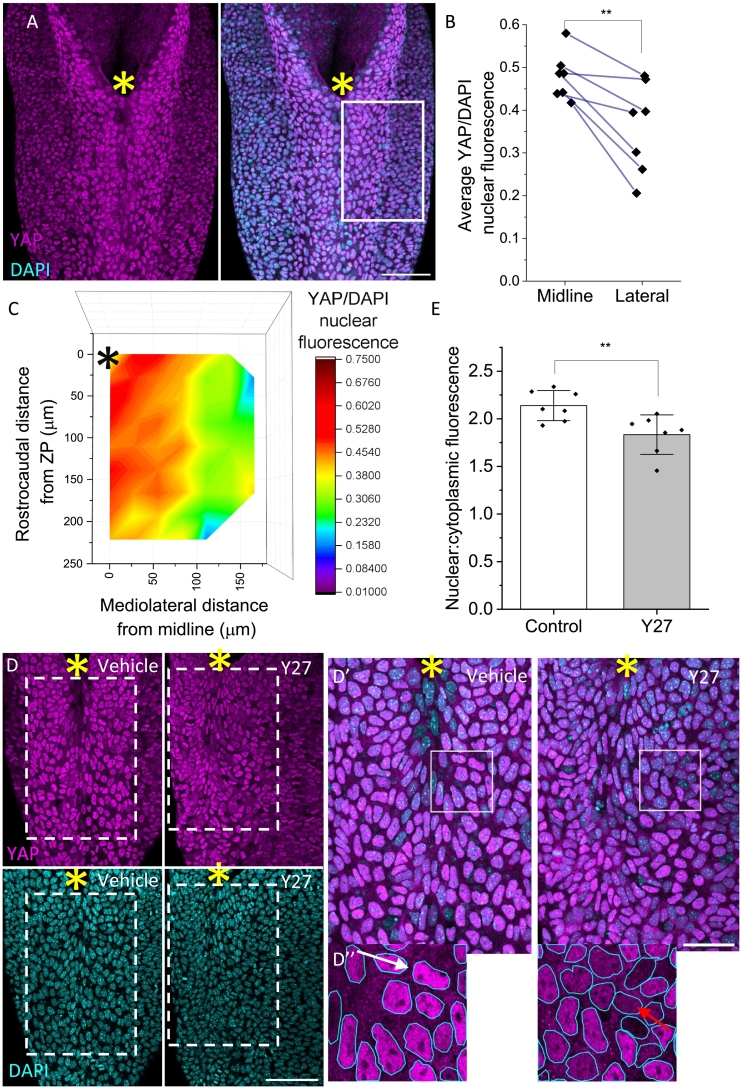
Surface ectoderm tension heterogeneity is reflected by YAP nuclear accumulation. A) YAP and DAPI whole mount immunofluorescence in wildtype E9.5 mouse embryos, with YAP only (left) and YAP and DAPI superimposed (right). Yellow asterisk = ZP. Scale bar = 100 μm. B) YAP/DAPI nuclear fluorescence reveals that nuclear accumulation of YAP is greater at the midline compared to laterally (** paired *t*-test, p < 0.006, n = 7 embryos). Blue lines connect data points from the same embryo. C) A significant negative gradient of YAP nuclear accumulation is revealed in a heatmap showing YAP/DAPI nuclear fluorescence (Pearson's correlation = − 0.27, p < 3 × 10–78, n = 4603 nuclei over 7 embryos). Data gathered from a region of SE as represented by white box in A. Data from both sides of the midline are included, but reflected together on one side, with the assumption that left and right sides are equal. Black asterisk = ZP. D) YAP and DAPI whole mount immunofluorescence in vehicle (left) and Y27 (right) treated embryos. Scale bar = 100 μm. D′) Zoomed images of white dashed boxed regions in D) with superimposed YAP and DAPI. Yellow asterisk = ZP. Scale bar = 50 μm. D″) Zoom of white boxes in D′) shows more intense nuclear compared to cytoplasmic YAP staining in vehicle controls (white arrow) than in Y27-treated embryos (red arrow). Nuclei outlined in cyan. E) Nuclear/cytoplasmic YAP fluorescence ratio is reduced in embryos culture for 8 h with Y27 (mean +/− SD, ** t-test, p < 0.01, n = 14 embryos).

As a cellular readout of mechanical stimuli, we analysed YAP nuclear translocation, which has previously been shown to characterise cells under greater tension (Fig. 4A) (Dupont et al., 2011). We explored spatial patterns of nuclear translocation by comparing YAP normalised to DAPI nuclear fluorescence. In agreement with the findings of ablation experiments, there is a significant mediolateral nuclear YAP gradient, with the midline showing higher nuclear YAP fluorescence compared to laterally (Figs. 4B–C, S6). This mediolateral gradient was independently confirmed in wild type embryos on a different background, BALB/c (Figs. S7A–B). We then asked whether the rostrocaudal regional difference in recoil revealed by laser ablation was similarly reflected by YAP expression. We took lower magnification images of C57/BL6 wild type embryos, as this enabled us to examine YAP expression from Zone 1 into Zone 2 (Fig. S7C). Indeed, a significant rostrocaudal gradient of YAP/DAPI fluorescence was observed, in agreement with our regional tension mapping (Fig. S7D). Of note, in all three experiments in wild type embryos, midline nuclei immediately at the ZP appear to have lower YAP levels despite the overall higher midline levels, in agreement with regional tension heterogeneity.

Finally, we asked whether YAP nuclear translocation could reflect abnormal biomechanics in a model where NTC is disrupted. Rho kinase (Rock) inhibition leads to a wider PNP, global reduction in tension, and diminished F-actin cables after 8 h of culture (Butler et al., 2019). We analysed YAP nuclear localisation in the SE of embryos treated with the Rock inhibitor Y27632, by comparing nuclear and cytoplasmic YAP fluorescence (Fig. 4D); a higher nuclear:cytoplasmic ratio is indicative of higher YAP activity. Strikingly, we showed that 8 h of Rock inhibition leads to a significant reduction in nuclear:cytoplasmic YAP (Fig. 4E), supporting our hypothesis that YAP nuclear translocation in the SE reflects cellular tension.

## 4 Discussion

The SE is increasingly recognised as an active driver of NTC through molecular and biomechanical contributions. Previous studies, in embryos carrying mutant alleles of *Grhl2*, demonstrated an association between dysregulation of multiple cell junction components, including components of tight junctions and adherens junctions, and failure of spinal NTC. Functional inhibition of claudins, which are tight junction components, also delays spinal NTC in mouse embryos (Baumholtz et al., 2017). However, the contribution of individual components of apical junction complexes in the SE to NT morphogenesis is not well defined (Nikolopoulou et al., 2019; Werth et al., 2010). Here, we showed that inhibition of adherens junctions by blocking E-cadherin is sufficient to alter NT morphology, with loss of the defined ZP and altered bending at DLHPs within the NE. These findings emphasise the impact of physical interactions between adjacent embryonic tissue layers, specifically the SE and NE.

As well as altering PNP morphology, blocking E-cadherin leads to a markedly disrupted F-actin cable around the PNP, suggesting an important role of adherens junctions in formation and/or maintenance of the cable. F-actin cables are evolutionarily conserved structures found at the leading edge of closing gaps, such as between the amnioserosa and epidermal cells in *Drosophila* dorsal closure, at the leading edge of wound healing events, and between the SE and the NE in the mammalian HNP (Maniou et al., 2021; Laplante and Nilson, 2011; Martin and Lewis, 1992). In most examples the cable surrounds the gap and is thought to promote closure by a purse string-like mechanism. However, a key difference of the PNP cable is that it does not encircle the PNP until the final stages of closure, when a rostrally directed closure 5 forms to complete the process (Galea et al., 2017). It is therefore unlikely that the PNP cable acts as a purse string, and may instead behave in conjunction with other pro-closure mechanisms such as DLHP formation, cell migration and NE apical constriction. In our E-cadherin blocking experiment, disruption of the cable is concurrent with altered morphology of the PNP, suggesting that the cable may directly contribute to PNP shaping, and is therefore involved in zippering as a secondary effect. Indeed, in *Grhl2* overexpressing mouse embryos, the PNP is notably narrow and the cable is abnormally abundant, while *Grhl2*^*−/−*^ embryos show a ZP curvature reminiscent of E-cadherin-inhibited embryos in this study, both in combination with a disrupted cable. In both *Grhl2* mutants the PNP length is significantly enlarged compared to wild type littermates, despite showing opposing cable phenotypes (Nikolopoulou et al., 2019). We speculate that, if we could culture our embryos for >24 h after treatment, E-cadherin blocked embryos would show inhibited ZP progression as a secondary effect to the enlarged ZP curvature, as in *Grhl2*^*−/−*^ embryos. How adherens junctions may be important in formation and/or maintenance of the F-actin cable itself is unclear. E-cadherin is known to both regulate and be regulated by actomyosin activity. For example, in mammary epithelial monolayers, Myosin-II accumulation at cell borders depends on E-cadherin activity, while Myosin-II inhibition disrupts E-cadherin distribution (Shewan et al., 2005). Similarly, during neurulation in *Ciona robusta*, Cadherin-2 activity directs localised myosin expression to the NE-SE border at the leading edge (Hashimoto and Munro, 2019). In conclusion, our results suggest that E-cadherin is involved in F-actin cable formation, and that in turn this is important for shaping of the PNP.

A key focus of this study was to investigate why midline SE cells are characteristically elongated in the region overlying the recently closed NT. Other models of gap closure events similarly show cell elongation in the direction of a closing edge, such as in *Drosophila* dorsal closure, *Xenopus* blastopore closure, zebrafish epiboly and wound healing (Galko and Krasnow, 2004; Koppen et al., 2006; Feroze et al., 2015). Force inference using static cell shape analysis is a common approach to predict the forces experienced by cells and tissues (Kong et al., 2018). In a recent study examining *Xenopus* NTC, Christodoulou and Skourides interpret SE cell shape at the leading edge to be a result of passive stretching by the NE (Christodoulou and Skourides, 2022). By increasing tension in the SE, they showed that NTC was disrupted, suggesting that low tension at the SE leading edge (comparable to Zone 1 in our study) in combination with force-generation by the NE is permissive to closure.

However, our study and others do not support passive stretching as a cause of cell elongation. In the current study we found that tension anisotropy cannot be predicted from cell shape, supporting the idea that the transient SE cell elongation at the ZP is not caused by passive stretching. A similar finding has been reported in *Drosophila*, whose epidermal cells are elongated in the direction of dorsal closure. This was also thought to result from passive stretching of the cells by active contraction of the neighbouring amnioserosa. However, by inducing contraction of leading-edge epidermis, and thereby eliminating cell elongation, Lv et al. showed that dorsal closure was disrupted, thus suggesting that cell elongation is an active process. Furthermore, single cell border tension at the leading edge is lower during active closure when compared to before closure, despite an overall increase in tissue tension (Lv et al., 2022). The authors suggested that structures which are not directly ablated, such as parts of the cytoplasm and the microtubule cytoskeleton, could be involved in bearing the increased tension, and indeed they found that microtubule dynamics were significantly rearranged in the elongated epidermal cells during closure compared to before (Lv et al., 2022). A similar mechanism has been observed in *Xenopus* neural crest cells, which decrease in stiffness just before migration due to a decrease in microtubule acetylation, a mechanism which is sufficient to initiate migration *in vitro* (Marchant et al., 2022). Microtubule dynamics could therefore be of interest for further study in SE neurulation, as they could be hypothesised to be responsible for reduced cell-border recoil.

The hypothesis that rostrocaudal elongation is caused by active cell behaviours rather than passive stretching is supported by live imaging in both the PNP and HNP (Mole et al., 2020; Maniou et al., 2021). *In silico* modelling of mouse HNP closure shows that medial crawling of leading edge cells is required in combination with F-actin cable activity (Maniou et al., 2021). In the PNP, formation of a semi-rosette around the ZP is dependent on anchorage of SE to an integrin-rich focal point at the ZP (Mole et al., 2020). This anchorage causes cells to shorten their proximal junctions and promotes their contact and fusion at the midline. Moreover, as the SE cells emerge rostrally from the ZP to overlie the most recently closed NT, they simultaneously adopt a rostrocaudally elongated morphology. This is achieved by constriction of the cells' distal ends to match the already constricted proximal ends, arguing for an active shape change in the SE cells at this point. Alteration of the semi-rosette pattern, when the focal point is genetically disrupted, is associated with failure of spinal NTC (Mole et al., 2020). Longer-term live imaging of both normal and failing closure will be important in further examining this hypothesis.

Finally, we show that YAP activity is regionally heterogenous, with higher nuclear expression levels in the midline compared to laterally. Although our cell border ablation data suggested that elongated cells are not under higher tension, there was a significant difference in recoil between midline and lateral cells in the most recently closed region, which correlates with the YAP nuclear intensity analysis. We also note that YAP midline translocation is similar to our observation of enriched midline expression of E-cadherin and the previously reported pattern of Claudin-4 expression (Nikolopoulou et al., 2019). In both *Drosophila* and mammals, multiple signalling complexes at AJ and septate/tight junctions have been shown to regulate Hippo signalling (Fulford et al., 2018). Because of the large number of regulatory networks involved, not much is known about how AJs in mammals and YAP/TAZ are linked. Some studies have found that E-cadherin-mediated contact inhibition downregulates YAP activity in order to control growth (Kim et al., 2011; Yang et al., 2015). Conversely, RNAi-mediated knockdown of E-cadherin and α-catenin in *Drosophila* imaginal disks causes cell autonomous reduction in Yki (*Drosophila* YAP) signalling and subsequent reduced tissue size (Yang et al., 2015). This suggests that nuclear translocation of YAP may be regulated by adherens/tight junction components and/or tension.

The most commonly discussed downstream function of YAP-activated transcription is in regulating cell growth, although YAP has a number of other diverse roles in development. Double knockout of LATS1/2 in mice leads to severe craniofacial malformations and cranial NTDs, as a result of increased YAP translocation to the nucleus and activation of transcription (Martinez Traverso et al., 2022). Interestingly, EMT genes involved in cell migration were upregulated in these mutants, triggering an increased migration of neural crest cells. In addition to the NE and neural crest cells, YAP has also been linked to migration in other contexts, suggesting it could play a role in a range of tissues including the SE. For example, YAP/TAZ activity regulates pro-migratory gene expression during mouse angiogenesis, and similarly Yki shows a pro-migratory role in border cell migration (Lucas et al., 2013; Wang et al., 2017). Whether YAP is involved in promoting migration of midline SE cells away from the ZP, and is therefore upstream of rostrocaudal elongation, will be an important question for future work.

In conclusion, this study demonstrates that SE mechanics can impact PNP morphology, including DLHPs in the adjacent NE. We show that cell shape in the recently closed NT is not caused by anisotropic tension, but that tension is regionally heterogenous leading to mechanical regulation of YAP signalling, thus providing a new example of YAP mechanical regulation during a crucial early developmental process.

## Funding

This study was funded by a Child Health Research CIO studentship (to ARM, NDEG) and the 10.13039/501100000265Medical Research Council (G0802163 to NDEG, AJC). GLG was funded by 10.13039/100010269Wellcome Trust grants 211112/Z/18/Z and 211112/Z/18/A.

## CRediT authorship contribution statement

**Abigail R. Marshall:** conceptualisation, methodology, investigation, writing, editing. **Gabriel L. Galea:** conceptualisation, investigation, editing. **Andrew J. Copp:** conceptualisation, methodology, editing. **Nicholas D.E. Greene:** conceptualisation, methodology, writing, editing.

## Declaration of competing interest

The authors declare no conflicts of interest.

## Appendix A Supplementary data

Supplementary figures Image 1

## References

[bb0005] Greene N.D., Copp A.J. (2014). Neural tube defects. Annu. Rev. Neurosci..

[bb0010] Nikolopoulou E. (2017). Neural tube closure: cellular, molecular and biomechanical mechanisms. Development.

[bb0015] Ybot-Gonzalez P. (2007). Neural plate morphogenesis during mouse neurulation is regulated by antagonism of bmp signalling. Development.

[bb0020] Rolo A. (2016). Regulation of cell protrusions by small GTPases during fusion of the neural folds. elife.

[bb0025] Ray H.J., Niswander L.A. (2016). Dynamic behaviors of the non-neural ectoderm during mammalian cranial neural tube closure. Dev. Biol..

[bb0030] Mole M.A. (2020). Integrin-mediated focal Anchorage drives epithelial zippering during mouse neural tube closure. Dev. Cell.

[bb0035] Galea G.L. (2021). Cell non-autonomy amplifies disruption of neurulation by mosaic Vangl2 deletion in mice. Nat. Commun..

[bb0040] Yu Z. (2006). The grainyhead-like epithelial transactivator Get-1/Grhl3 regulates epidermal terminal differentiation and interacts functionally with LMO4. Dev. Biol..

[bb0045] Rifat Y. (2010). Regional neural tube closure defined by the grainy head-like transcription factors. Dev. Biol..

[bb0050] Brouns M.R. (2011). Over-expression of Grhl2 causes spina bifida in the axial defects mutant mouse. Hum. Mol. Genet..

[bb0055] Simpson C.L., Patel D.M., Green K.J. (2011). Deconstructing the skin: cytoarchitectural determinants of epidermal morphogenesis. Nat Rev Mol Cell Biol.

[bb0060] Kenny F.N. (2018). Tissue stiffening promotes keratinocyte proliferation through activation of epidermal growth factor signaling. J. Cell Sci..

[bb0065] Zhang H., Pasolli H.A., Fuchs E. (2011). Yes-associated protein (YAP) transcriptional coactivator functions in balancing growth and differentiation in skin. Proc. Natl. Acad. Sci. U. S. A..

[bb0070] Totaro A. (2017). YAP/TAZ link cell mechanics to notch signalling to control epidermal stem cell fate. Nat. Commun..

[bb0075] Sedov E. (2022). THY1-mediated mechanisms converge to drive YAP activation in skin homeostasis and repair. Nat. Cell Biol..

[bb0080] Dupont S. (2011). Role of YAP/TAZ in mechanotransduction. Nature.

[bb0085] Kono K., Tamashiro D.A., Alarcon V.B. (2014). Inhibition of RHO-ROCK signaling enhances ICM and suppresses TE characteristics through activation of hippo signaling in the mouse blastocyst. Dev. Biol..

[bb0090] Escuin S. (2015). Rho-kinase-dependent actin turnover and actomyosin disassembly are necessary for mouse spinal neural tube closure. J. Cell Sci..

[bb0095] Galea G.L. (2017). Biomechanical coupling facilitates spinal neural tube closure in mouse embryos. Proc. Natl. Acad. Sci. U. S. A..

[bb0100] Nikolopoulou E. (2019). Spinal neural tube closure depends on regulation of surface ectoderm identity and biomechanics by Grhl2. Nat. Commun..

[bb0105] Maniou E. (2021). Hindbrain neuropore tissue geometry determines asymmetric cell-mediated closure dynamics in mouse embryos. Proc. Natl. Acad. Sci. U. S. A..

[bb0110] Butler M.B. (2019). Rho kinase-dependent apical constriction counteracts M-phase apical expansion to enable mouse neural tube closure. J. Cell Sci..

[bb0115] Baumholtz A.I. (2017). Claudins are essential for cell shape changes and convergent extension movements during neural tube closure. Dev. Biol..

[bb0120] Wang C. (2014). Glial cell-derived neurotrophic factor attenuates neuropathic pain in a mouse model of chronic constriction injury: possible involvement of E-cadherin/p120ctn signaling. J. Mol. Neurosci..

[bb0125] Nakagawa M. (2001). Recruitment and activation of Rac1 by the formation of E-cadherin-mediated cell-cell adhesion sites. J. Cell Sci..

[bb0130] Marshall A.R. (2022). Two-photo cell and tissue level laser ablation methods to study morphogenetic biomechanics. Methods Mol. Biol..

[bb0135] Galea G.L. (2018). Vangl2 disruption alters the biomechanics of late spinal neurulation leading to spina bifida in mouse embryos. Dis. Model. Mech..

[bb0140] Aigouy B., Umetsu D., Eaton S. (2016). Segmentation and quantitative analysis of epithelial tissues. Methods Mol. Biol..

[bb0145] Legland D., Arganda-Carreras I., Andrey P. (2016). MorphoLibJ: integrated library and plugins for mathematical morphology with ImageJ. Bioinformatics.

[bb0150] Bolte S., Cordelières F.P. (2006). A guided tour into subcellular colocalization analysis in light microscopy. J. Microsc..

[bb0155] Tseng Q. (2012). Spatial organization of the extracellular matrix regulates cell-cell junction positioning. Proc. Natl. Acad. Sci. U. S. A..

[bb0160] Werth M. (2010). The transcription factor grainyhead-like 2 regulates the molecular composition of the epithelial apical junctional complex. Development.

[bb0165] Laplante C., Nilson L.A. (2011). Asymmetric distribution of echinoid defines the epidermal leading edge during drosophila dorsal closure. J. Cell Biol..

[bb0170] Martin P., Lewis J. (1992). Actin cables and epidermal movement in embryonic wound healing. Nature.

[bb0175] Shewan A.M. (2005). Myosin 2 is a key rho kinase target necessary for the local concentration of E-cadherin at cell-cell contacts. Mol. Biol. Cell.

[bb0180] Hashimoto H., Munro E. (2019). Differential expression of a classic cadherin directs tissue-level contractile asymmetry during neural tube closure. Dev. Cell.

[bb0185] Galko M.J., Krasnow M.A. (2004). Cellular and genetic analysis of wound healing in drosophila larvae. PLoS Biol..

[bb0190] Koppen M. (2006). Coordinated cell-shape changes control epithelial movement in zebrafish and drosophila. Development.

[bb0195] Feroze R. (2015). Mechanics of blastopore closure during amphibian gastrulation. Dev. Biol..

[bb0200] Kong W. (2018).

[bb0205] Christodoulou N., Skourides P.A. (2022). Distinct spatiotemporal contribution of morphogenetic events and mechanical tissue coupling during xenopus neural tube closure. Development.

[bb0210] Lv Z. (2022). The lateral epidermis actively counteracts pulling by the amnioserosa during dorsal closure. Front. Cell. Dev. Biol..

[bb0215] Marchant C.L. (2022). Cell clusters softening triggers collective cell migration in vivo. Nat. Mater..

[bb0220] Fulford A., Tapon N., Ribeiro P.S. (2018). Upstairs, downstairs: spatial regulation of hippo signalling. Curr. Opin. Cell Biol..

[bb0225] Kim N.G. (2011). E-cadherin mediates contact inhibition of proliferation through hippo signaling-pathway components. Proc. Natl. Acad. Sci. U. S. A..

[bb0230] Yang C.C. (2015). Differential regulation of the hippo pathway by adherens junctions and apical-basal cell polarity modules. Proc. Natl. Acad. Sci. U. S. A..

[bb0235] Martinez Traverso I.M. (2022). LATS1/2 control TGFB-directed epithelial-to-mesenchymal transition in the murine dorsal cranial neuroepithelium through YAP regulation. Development.

[bb0240] Lucas E.P. (2013). The hippo pathway polarizes the actin cytoskeleton during collective migration of drosophila border cells. J. Cell Biol..

[bb0245] Wang X. (2017). YAP/TAZ orchestrate VEGF signaling during developmental angiogenesis. Dev. Cell.

