# Supplementary material for: The surface ectoderm exhibits spatially heterogenous tension that correlates with YAP localisation during spinal neural tube closure in mouse embryos
Source: Cells Dev. 2023 Jun;174:None. doi: 10.1016/j.cdev.2023.203840 (PMC10618430; doi:10.1016/j.cdev.2023.203840)
Supplement: Supplementary file 1 — Supplementary figures [file mmc1.pdf]

Figure S1

Midline SE cells are rostrocaudally elongated and oriented towards the midline.

A) The shapes of cells touching a line drawn along the midline (yellow dashed line) of the analysis region (white box) are compared to those at the lateral boundary of the analysis region. Yellow asterisk = ZP. B) Midline SE cells are significantly more elongated compared to lateral cells (mean +/- SD, * paired t-test, $p < 0.02$, n = 8 embryos). C) Midline cells are significantly more oriented towards the rostrocaudal axis (mean +/- SD, *** paired t-test, $p < 0.001$, n =8 embryos). D, E) Heat maps showing aspect ratio and orientation of cells according to their position relative to the ZP (0,0). The X and Y axes represent the mediolateral and rostrocaudal axes respectively. Data points are all reflected onto one side of the midline, with the assumption that the left and right sides are equal. Red asterisk = ZP. n = 591 cells over 8 embryos.

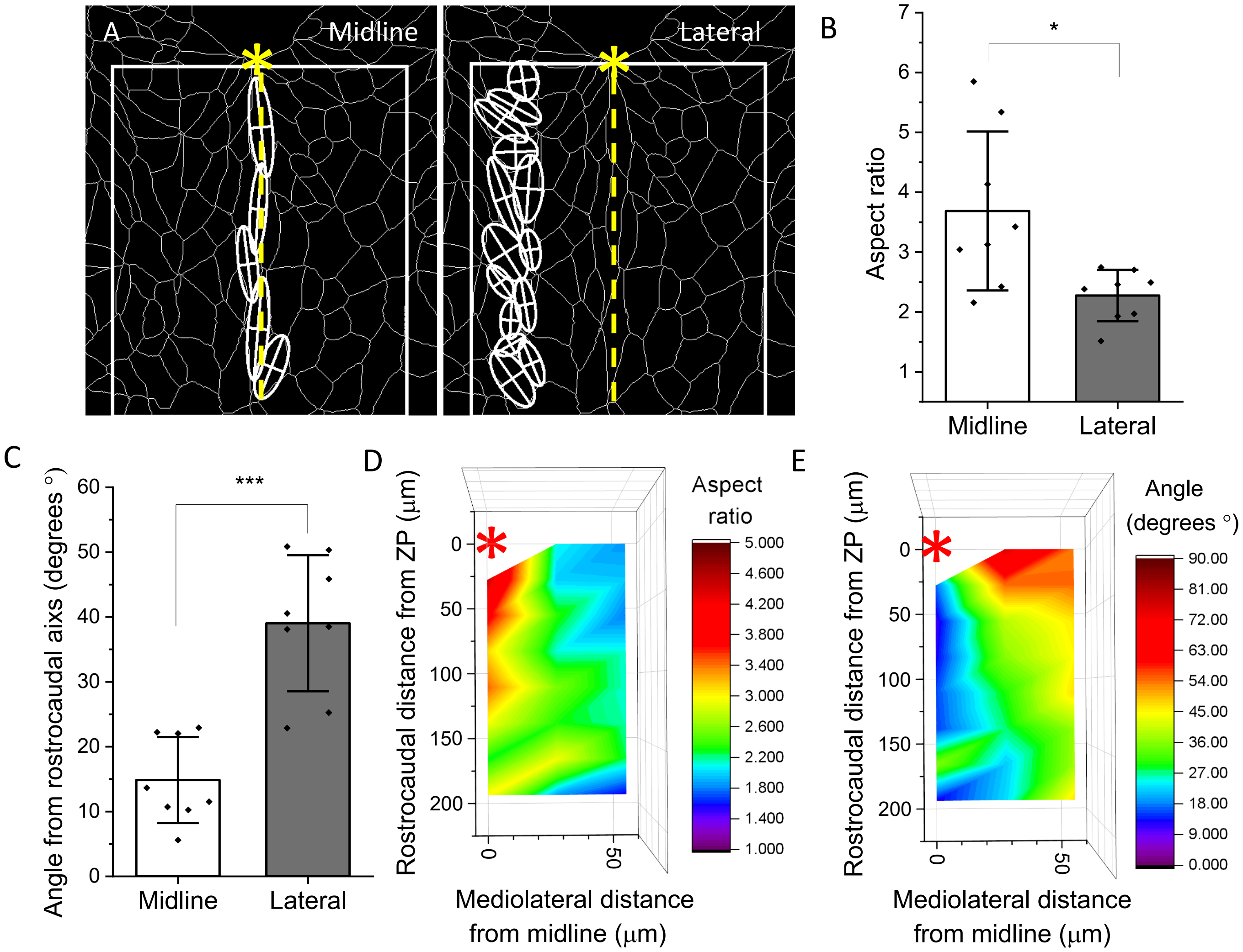

A
Midline
Lateral
B
Aspect ratio
*
Midline
Lateral
C
Angle from rostrocaudal aixs (degrees °)
***
Midline
Lateral
D
Rostrocaudal distance from ZP (μm)
Mediolateral distance from midline (μm)
Aspect ratio
5.000
4.600
4.200
3.800
3.400
3.000
2.600
2.200
1.800
1.400
1.000
E
Rostrocaudal distance from ZP (μm)
Mediolateral distance from midline (μm)
Angle (degrees °)
90.00
81.00
72.00
63.00
54.00
45.00
36.00
27.00
18.00
9.000
0.000

Figure S2

E cadherin is expressed in the SE and not the NE. Endogenous E-cadherin-detecting antibody and E-cadherin-blocking antibody show distinct but overlapping expression patterns.

A) Reslice of E9.5 embryo in Figure 1A. A cross section through the image at the ZP shows E-cadherin (magenta) is expressed specifically in the SE (yellow arrow) but not the NE (red arrow). Scale bar = 100 µm. B – D) Magnified images of cells from Figure 1B in the midline (B, C) and lateral (D) of control (B) or embryos treated with blocking antibody (C, D). Immunofluorescence was used to visualise the blocking antibody (magenta) and endogenous E-cadherin (green). At the midline of embryos cultured with blocking antibody (C), both antibodies can be visualised at the cell border, and the blocking antibody can also be visualised throughout the cytoplasm. Laterally (D), a similar staining pattern is observed, but levels of endogenous E-cadherin are lower. Scale bars = 25 µm.

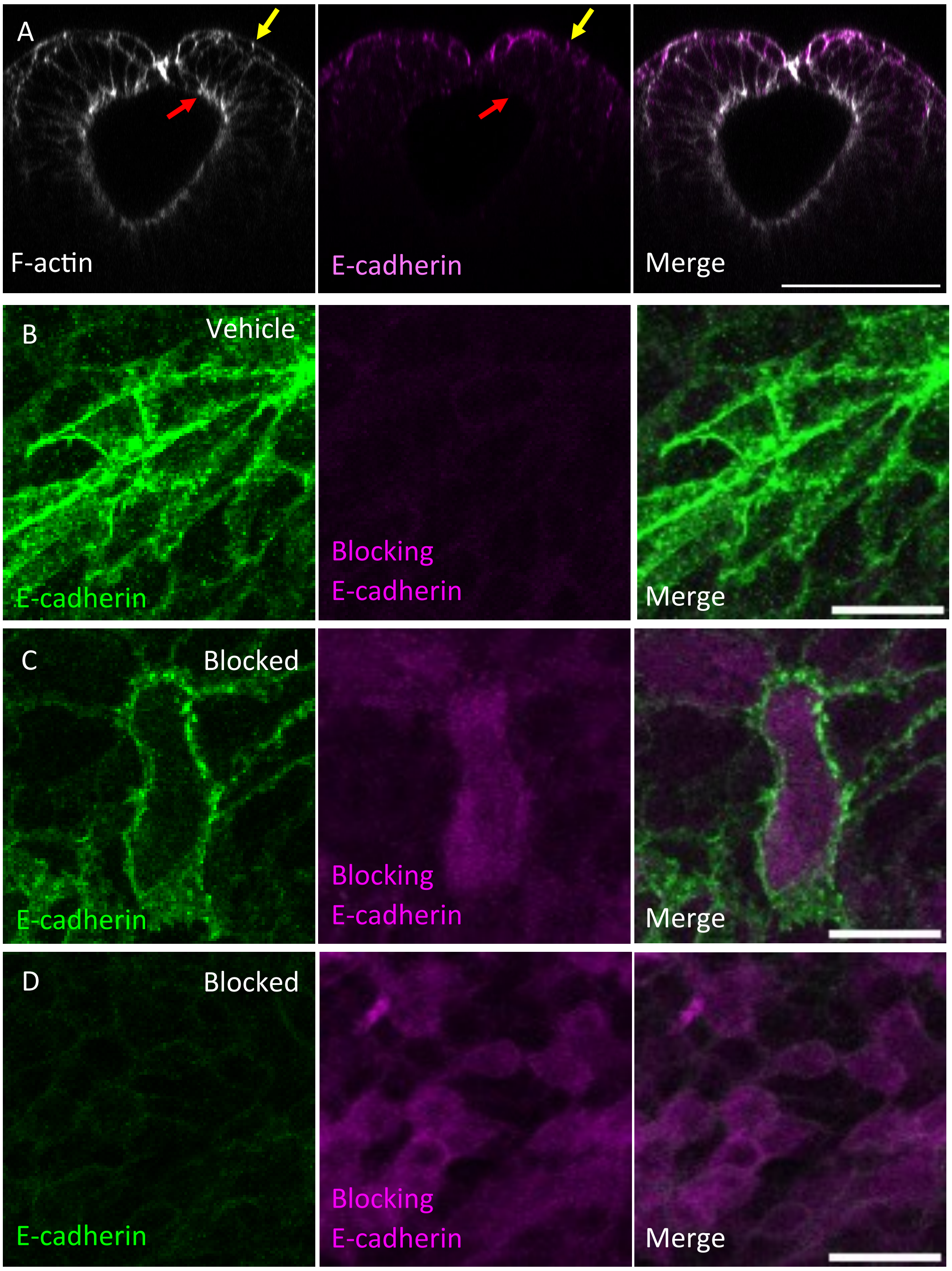
A
F-actin
E-cadherin
Merge
B
Vehicle
E-cadherin
Blocking
E-cadherin
Merge
C
Blocked
E-cadherin
Blocking
E-cadherin
Merge
D
Blocked
E-cadherin
Blocking
E-cadherin
Merge

Figure S3

E-cadherin blocking antibody does not affect embryo somite gain or PNP length, but causes significant distruption to the F-actin cable. Embryos with a more severely disrupted curvature also have a more severely disrupted DLHP angle.

A) Somite gain over a culture period of 24 hours (as measured by counting somites at the end of culture) is comparable between controls (including non-injected and vehicle-injected) and embryos cultured with 1:200 blocking antibody (paired t-test, $p = 0.81$, $n = 9$ litters). Each point is the average of embryos in the same group from the same experiment. Connecting lines indicate embryos from the same litter (and therefore the same experiment). B) F-actin cable intensity was analysed by taking the fluorescence profile of a mediolaterally-oriented line drawn at the ZP (yellow dashed line). Scale bar = 100 µm. C) Peak intensity was normalised to the average fluorescence intensity of a profile drawn through the ZP, and compared between controls and embryos cultured with 1:200 blocking antibody (*t-test, $p < 0.02$, $n = 22$ embryos). D) There is no significant difference in PNP length between controls and E-cadherin blocking antibody treated embryos at either somite stage analysed (17-19 somites, Mann-Whitney test, $p = 0.4$, $n = 23$ embryos; 20-22 somites, t-test, $p = 0.25$, $n = 21$ embryos). E) In embryos cultured with 1:200 E-cadherin blocking antibody, embryos with a larger curvature also showed a smaller DLHP angle (linear regression, $r^2 = 0.29$, F-test, $p < 0.02$, $n = 20$ embryos).

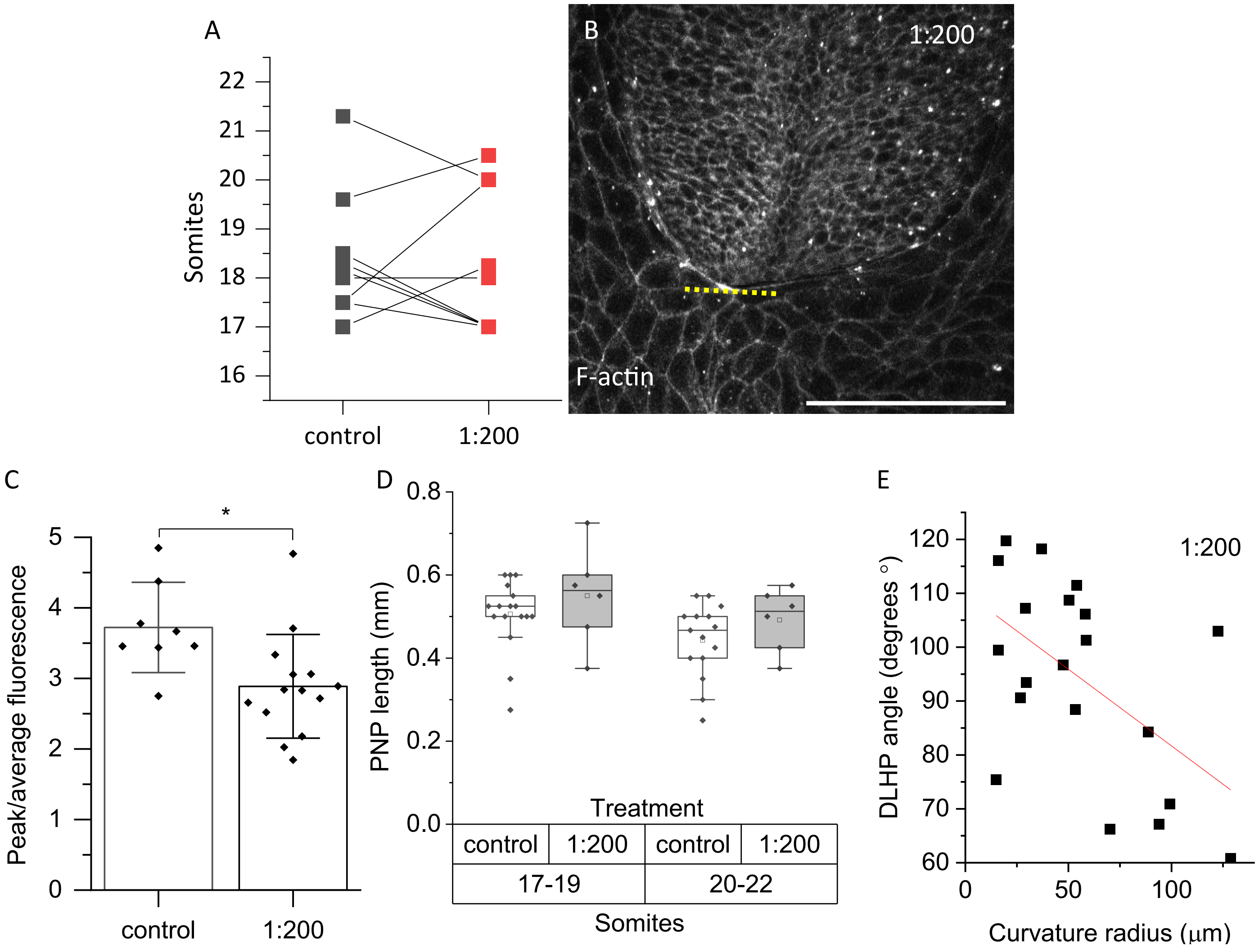

A
Somites
22
21
20
19
18
17
16
control
1:200
B
1:200
F-actin
C
*
Peak/average fluorescence
5
4
3
2
1
0
control
1:200
D
PNP length (mm)
0.8
0.6
0.4
0.2
0.0
Treatment
control
1:200
control
1:200
17-19
20-22
Somites
E
DLHP angle (degrees °)
1:200
120
110
100
90
80
70
60
0
50
100
Curvature radius (μm)

Figure S4

SE ablations in wild type and *Grhl2*$^{-/-}$ cells.

A, B) Example ablations of wild type (A) and *Grhl2*$^{-/-}$ (B) SE cells in the region rostral to the ZP. Images show before (A, B), after (A', B') and composite (A'', B''). White circles indicate landmarks used to analyse recoil. Scale bars = 10 µm.

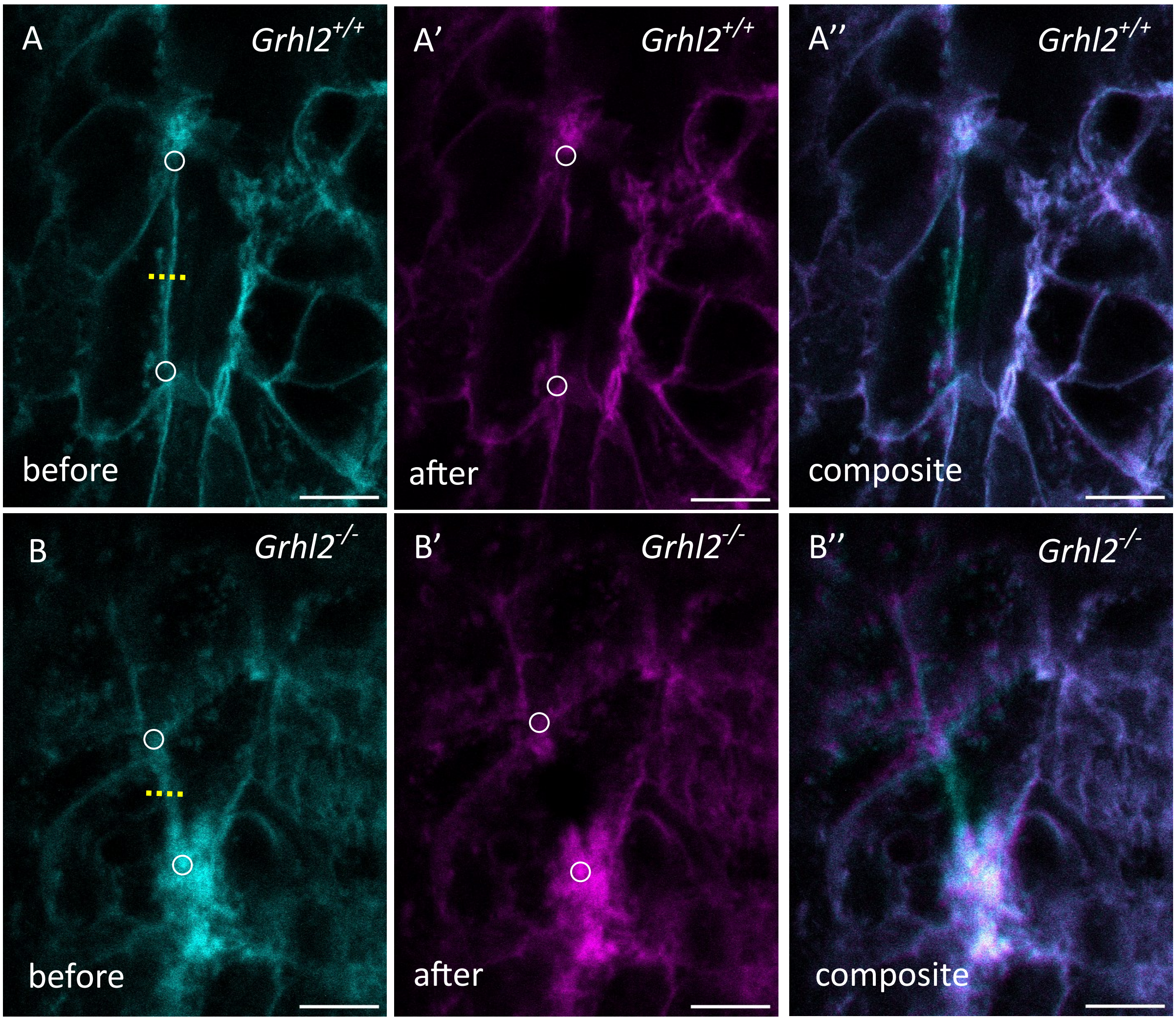
A
Grhl2+/+
before
A'
Grhl2+/+
after
A''
Grhl2+/+
composite
B
Grhl2-/-
before
B'
Grhl2-/-
after
B''
Grhl2-/-
composite

Figure S5

Rostrocaudal recoil in the recently closed NT does not change with developmental stage.

Quantification of rostrocaudal recoil after single border laser ablation shows no difference in recoil between somite groups (ANOVA, $p = 0.91$, $n = 17$ embryos).

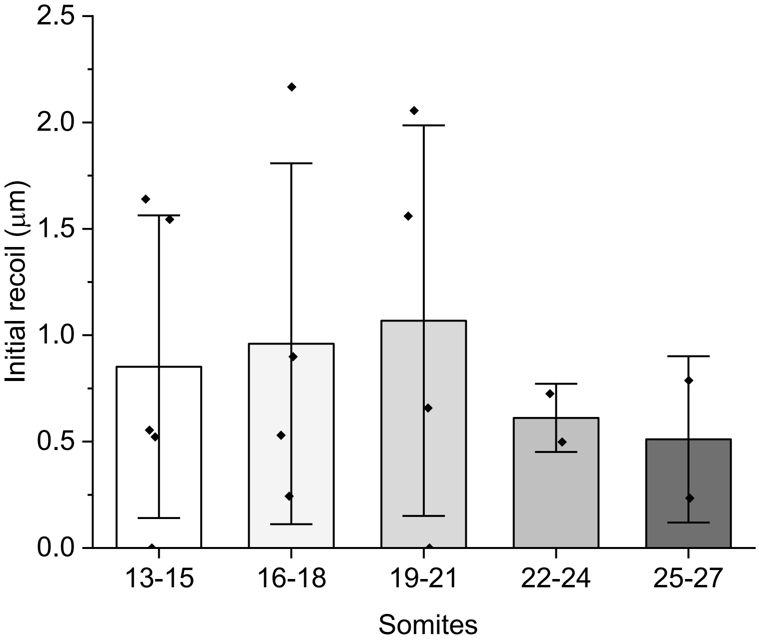

2.5
2.0
1.5
1.0
0.5
0.0
Initial recoil (μm)
13-15
16-18
19-21
22-24
25-27
Somites

Figure S6

The mediolateral gradient of YAP nuclear accumulation is consistent between embryos.

Box plot showing the mediolateral gradient of YAP/DAPI nuclear fluorescence by embryo (E1 – E7). Within each embryo, boxes represent regions of 100 pixels, from the midline (box 0) to the furthest lateral (up to 500, depending on embryo). Black lines connect the median of each box. n = 4603 nuclei over 7 embryos.

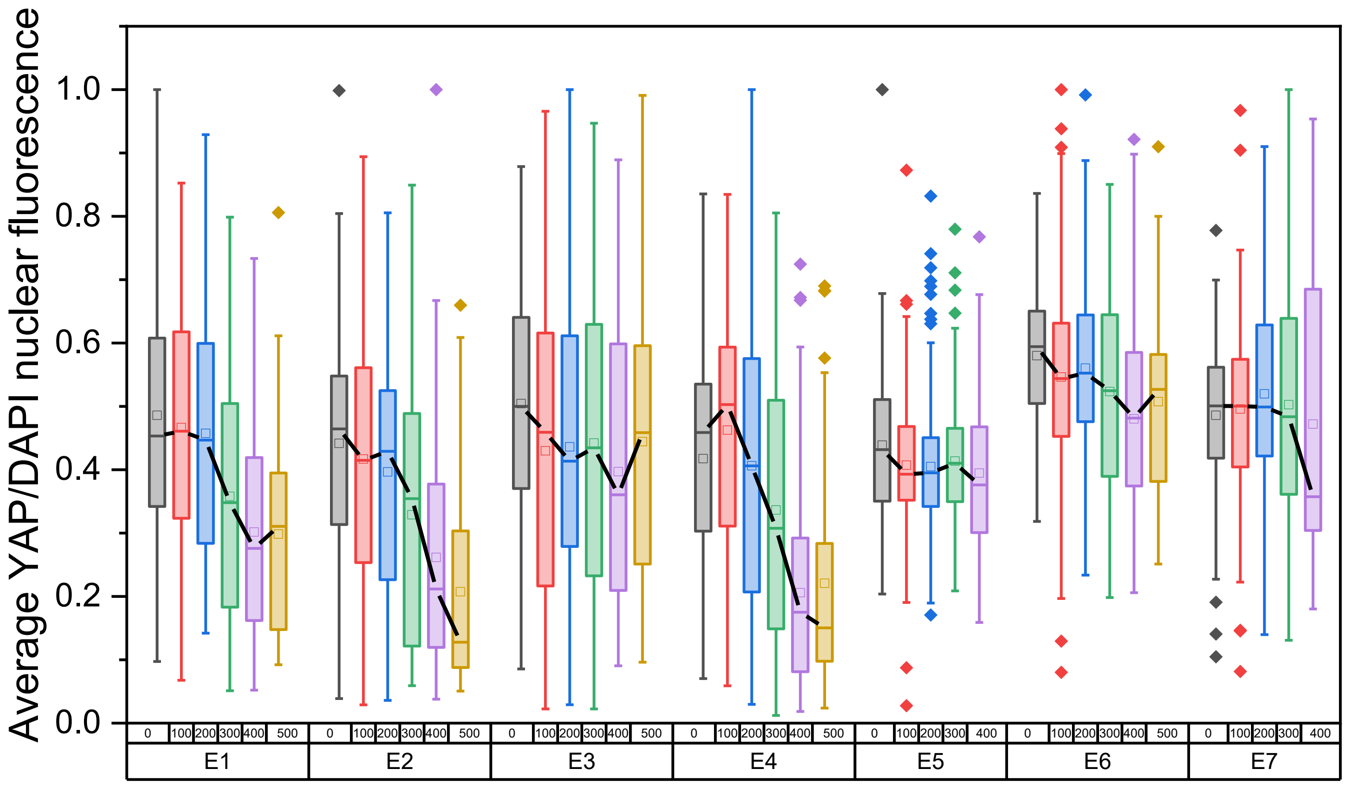
Average YAP/DAPI nuclear fluorescence
1.0
0.8
0.6
0.4
0.2
0.0
0 100 200 300 400 500
0 100 200 300 400 500
0 100 200 300 400 500
0 100 200 300 400 500
0 100 200 300 400
0 100 200 300 400 500
0 100 200 300 400
E1
E2
E3
E4
E5
E6
E7

Figure S7

YAP nuclear translocation reflects mediolateral and rostrocaudal tension heterogeneity.

A) YAP and DAPI whole mount immunofluorescence in wild type BALB/c E9.5 mouse embryos. Yellow asterisk = ZP. Scale bar = 100 µm. B) In the ZP region (Zone 1), a significant negative mediolateral gradient of YAP nuclear accumulation is revealed in a heatmap showing YAP/DAPI nuclear fluorescence (Pearson's correlation = - 0.27, $p < 4 \times 10^{-56}$, n = 3294 nuclei over 5 BALB/c embryos). Data from both sides of the midline are included, but reflected together on one side, with the assumption that left and right sides are equal. Black asterisk = ZP. C) YAP and DAPI whole mount immunofluorescence in wild type E9.5 C57/BL6 mouse embryos, with YAP only (left), DAPI only (middle) and YAP and DAPI superimposed (right). White box = region analysed in D). Yellow asterisk = ZP. Scale bar = 100 µm. D) A significant positive caudal-rostral gradient of YAP nuclear accumulation is revealed in a heatmap showing YAP/DAPI nuclear fluorescence (Pearson's correlation = 0.132, $p < 6 \times 10^{-20}$, n = 4759 nuclei over 4 C57/BL6 embryos). Black asterisk = ZP.

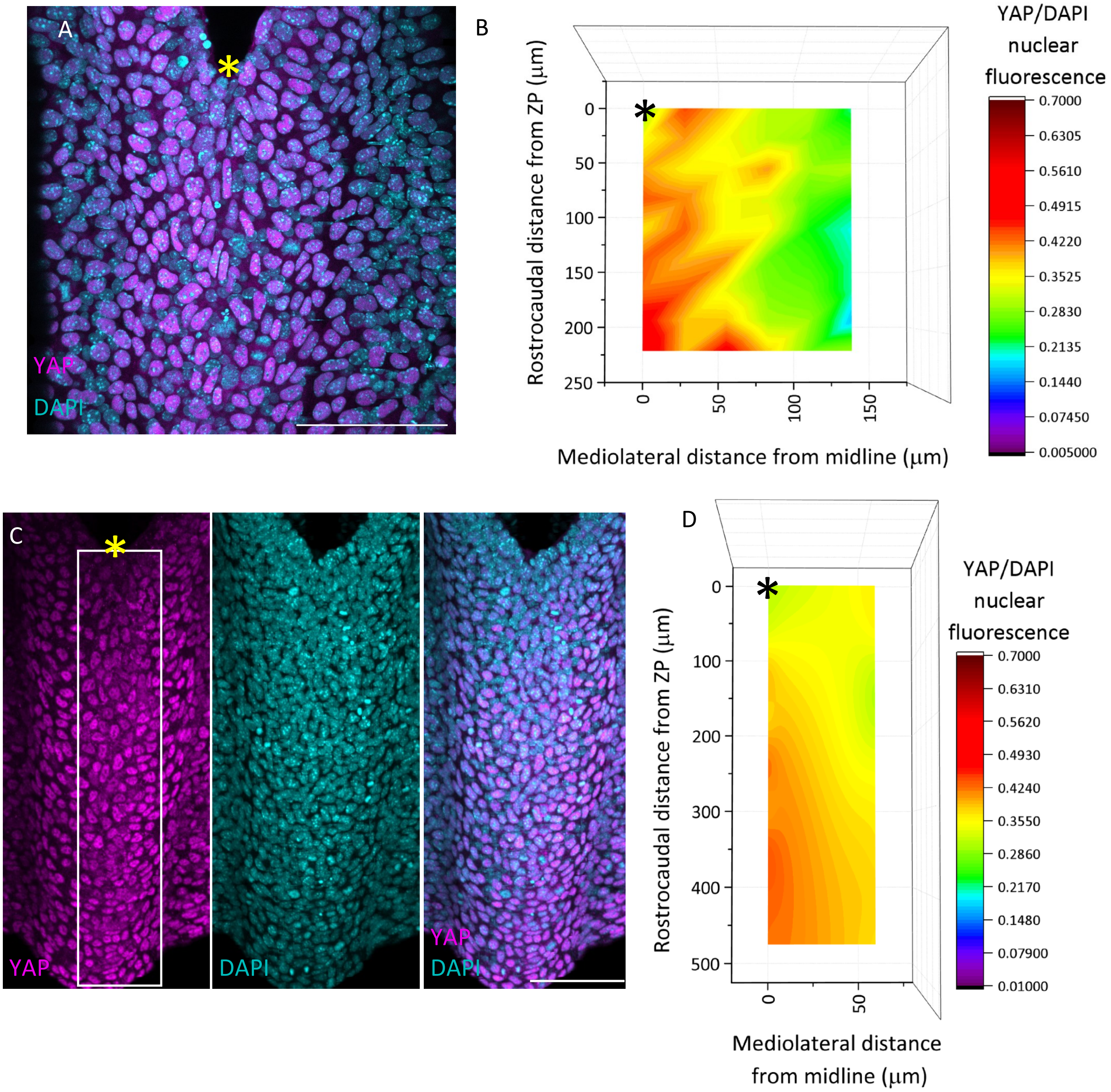
A
YAP
DAPI
B
YAP/DAPI nuclear fluorescence
Rostrocaudal distance from ZP (μm)
Mediolateral distance from midline (μm)
0.7000
0.6305
0.5610
0.4915
0.4220
0.3525
0.2830
0.2135
0.1440
0.07450
0.005000
C
YAP
DAPI
YAP
DAPI
D
YAP/DAPI nuclear fluorescence
Rostrocaudal distance from ZP (μm)
Mediolateral distance from midline (μm)
0.7000
0.6310
0.5620
0.4930
0.4240
0.3550
0.2860
0.2170
0.1480
0.07900
0.01000
